# Five new species, two new sexual morph reports, and one new geographical record of *Apiospora* (Amphisphaeriales, Sordariomycetes) isolated from bamboo in Yunnan, China

**DOI:** 10.3389/fcimb.2024.1476066

**Published:** 2024-12-11

**Authors:** Li-Su Han, Chao Liu, Dong-Qin Dai, Itthayakorn Promputtha, Abdallah M. Elgorban, Salim Al-Rejaie, Qiang Li, Nalin N. Wijayawardene

**Affiliations:** ^1^ Center for Yunnan Plateau Biological Resources Protection and Utilization, College of Biological Resource and Food Engineering, Qujing Normal University, Qujing, China; ^2^ Department of Biology, Faculty of Science, Chiang Mai University, Chiang Mai, Thailand; ^3^ Department of Botany and Microbiology, College of Science, King Saud University, Riyadh, Saudi Arabia; ^4^ Department of Pharmacology and Toxicology, College of Pharmacy, King Saud University, Riyadh, Saudi Arabia; ^5^ Tropical Microbiology Research Foundation, Pannipitiya, Sri Lanka

**Keywords:** Apiosporaceae, phylogeny, saprobes, taxonomy, bambusicolous fungi

## Abstract

*Apiospora* is an important genus in the Apiosporaceae family with a worldwide distribution. They exhibit different lifestyles including pathogenic, saprophytic, and endophytic. In this study, we aimed to explore the *Apiospora* associated with bamboo and collected 14 apiospora-like taxa from the forests of Yunnan Province, China. Morphological and phylogenetic analyses (combined ITS, LSU, *tef*1-*α*, and *tub*2 sequence data) confirmed that these collections belong to Apiospora s. str. and reports five new species (viz., *Ap. dehongensis, Ap. jinghongensis, Ap. shangrilaensis, Ap. zhaotongensis*, and *Ap. zhenxiongensis*). New sexual morphs of asexually typified *Ap. globose* and *Ap. guangdongensis* species, and a new geographical record of *Ap. subglobosa* are also reported. The findings of this study not only enhance the diversity of bambusicolous fungi in the region of Yunnan, but also geographical distribution of some known *Apiospora* species.

## Introduction

Apiosporaceae (Amphisphaeriales, Sordariomycetes, Ascomycota *fide* Wijayawardene et al., 2022) was introduced by Hyde et al. (1998), with *Apiospora* Sacc. as the type genus (Saccardo, 1875). Currently, Apiosporaceae comprises three genera—*Apiospora*, *Arthrinium* Kunze, and *Nigrospora* Zimm (Pintos and Alvarado, 2021; Jiang et al., 2022a). The relationship among the genera in Apiosporaceae is confusing as some taxa lack sequence data and show morphological plasticity. For example, *Apiospora* resembles *Arthrinium s. str.* but is phylogenetically well-distinct (Pintos and Alvarado, 2021; Jiang et al., 2022a).

The genus *Apiospora* was introduced by Saccardo (1875), with *Ap. montagnei* Sacc. as the type species (Clements and Shear, 1931). Currently, 175 epithets are listed under the genus of *Apiospora* in Index Fungorum (Index Fungorum 2024; accession date: 16 June 2024). *Apiospora* was reported as a holomorphic genus; the sexual morph is characterized by multi-locular stromata and 1-septate (near the lower cell) ascospores (Dai et al., 2016, 2017; Bhunjun et al., 2022). The asexual morphs usually occur as coelomycetous on natural substrates or hyphomycetous on culture (e.g., MEA, OA, and PDA). The coelomycetous morph is characterized by dark brown conidia, with a longitudinal and transparent slit (Dai et al., 2016; Pintos and Alvarado, 2021; Zhao et al., 2023). The hyphomycetous morph is characterized by hyaline conidiophores, basauxic conidiogenous cells, globose to subglobose, and pale brown to brown conidia (Wang et al., 2018; Pintos and Alvarado, 2021; Tian et al., 2021; Zhao et al., 2023).

Members of *Apiospora* are found in different habitats such as animal tissues (including humans), air, lichens, plants, soil, and seaweeds (Liao et al., 2023). The members of *Apiospora* show a wide distribution and have been reported from tropical, sub-tropical, Mediterranean, and temperate regions (Pintos and Alvarado, 2021; Kwon et al., 2022). Furthermore, the species of *Apiospora* have been reported as endophytes, saprobes, or pathogens, and some particular species have two or three lifestyles (Samuels et al., 1981; Liao et al., 2023; Zeng et al., 2024). For instance, *Ap. arundinis* (Corda) Pintos & P. Alvarado has been reported as pathogens, saprobes, and endophytes (Feng et al., 2021; Liao et al., 2023). Moreover, *Ap. arundinis* has also been reported as a pathogen of plants, animals, and humans (Martínez-Cano et al., 1992; Mavragani et al., 2007; Bagherabadi et al., 2014; Chen et al., 2014; Jiang and Tian, 2021).

According to Monkai et al. (2022); Zhao et al. (2023), and Liu et al. (2024), asexual morphs of *Apiospora* are frequently observable, while sexual morphs are rare. Therefore, this study aims to explore the morphology of the sexual morph of *Apiospora.* Of this, a total of 14 *Apiospora* samples were collected from the Yunnan Province, China. Among these new collections, five new species (e.g., *Ap. dehongensis*, *Ap. jinghongensis*, *Ap. shangrilaensis*, *Ap. zhaotongensis*, and *Ap. zhenxiongensis*), two sexual morphs of asexually typified species (*Ap. globosa* and *Ap. guangdongensis*), and one new country record (*Ap. subglobosa*) are reported along with the morphological descriptions, illustrations, and updated phylogenetic trees.

## Materials and methods

### Collection and morphological studies

The dead and decaying bamboo culms and branches were collected from several forests in Dehong, Shangri-La, Xishuangbanna, and Zhaotong in Yunnan Province. Collected samples were kept in envelope bags and transported to the lab for further evaluation. The stromata and micro-morphological characteristics were observed and photographed using Leica S8AP0 and Olympus BX53 stereomicroscopes, respectively, which are equipped with a high-definition digital camera. The sizes of the fungal structures were measured by the Tarosoft (R) Image Frame Work program (IFW). The photo plates were processed with the Adobe Photoshop CS6 software (Adobe Systems Inc., San Jose, CA, USA).

### Isolation and preservation

Pure cultures of the new collections were obtained by single spore isolation. The ascospores were picked from stromata and dispersed on sterile water droplets on a cavity slide. Spore suspensions were placed on potato dextrose agar (PDA) and stored at 27°C until germination. The germinated spores were aseptically transferred into new PDA plates and incubated. The characteristics of fungi colonies were recorded and photographed after 20 days.

Dried herbarium samples were preserved at the Mycological Herbarium of Zhongkai University of Agriculture and Engineering (MHZU) and Herbarium of Guizhou Medical University, Guiyang, China (GMB-W). Living cultures were deposited in Zhongkai University of Agriculture and Engineering (ZHKUCC) and Guizhou Medical University Culture Collection (GMBCC) Guiyang.

### DNA extraction, PCR, and sequencing

The genomic DNA was extracted from fresh fungal mycelia grown on PDA [using Biospin Fungus Genomic DNA Extraction Kit (BioFlux^®^)]. However, for two species, single spore isolation was not successful. Thus, we used fruiting bodies to extract DNA using an E.Z.N.A. Forensic DNA Kit (BIO-TEK). The details of the primers used for PCR amplification are presented in **Table 1**. We followed Tian et al. (2021) for PCR amplification conditions. PCR products were sequenced at Shanghai Mayobio Biomedical Technology Co., China. All newly generated nucleotide sequence data were submitted to GenBank and the accession numbers were obtained (**Table 2**).

**Table 1:** LSU, ITS, *tef*1-*α*, and *tub*2 loci primers information.

Loci	Primers and base pairs (5′ to 3′)	References
LSU (large subunit rDNA)	Forward: LROR 5′-GTACCCGCTGAACTTAAGC-3′ Reverse: LR5 5′-ATCCTGAGGGAAACTTC-3′	Vilgalys and Hester (1990)
ITS (internal transcribed spacers)	Forward: ITS5 5′-TCCTCCGCTTATTGATATGC-3′ Reverse: ITS4 5′-GGAAGTAAAAGTCGTAACAAGG-3′	White et al. (1990)
*tef*1-*α* (elongation factor 1-alpha)	Forward: EF1-728F 5′-CATCGAGAAGTTCGAGAAGG-3′ Reverse: EF-2 5′-GGARGTACCAGTSATCATGTT-3′	O'Donnell et al. (1998); Carbone and Kohn (1999)
*tub*2 (β-tubulin)	Forward: Bt2a 5′-GGTAACCAAATCGGTGCTGCTTTC-3′ Reverse: Bt2b 5′-ACCCTCAGTGTAGTGACCCTTGGC-3′	Glass and Donaldson (1995)

**Table 2:** Details of the taxa used in the phylogenetic analyses.

Taxa	Strains	Substrate	Country	Lifestyles	GenBank Accession Numbers			
					LSU	ITS	*tub*2	*tef*1-*α*
*Apiospora acutiapica*	KUMCC 20-0210^T^	*Bambusa bambos*	China	Saprobe	MT946339	MT946343	MT947366	MT947360
*Ap. adinandrae*	SAUCC 1282B-1^T^	*Adinandra glischroloma*	China	–	OR739572	OR739431	OR757128	OR753448
*Ap. agari*	KUC21333^T^	*Agarum cribrosum*	Republic of Korea	–	MH498440	MH498520	MH498478	MH544663
*Ap. aquatica*	S 642^T^	Submerged wood	China	Saprobe	MK835806	MK828608	NA	NA
*Ap. arctoscopi*	KUC21331^T^	Egg of *Arctoscopus japonicus*	Republic of Korea	–	MH498449	MH498529	MH498487	MN868918
*Ap. armeniaca*	SAUCC DL1831^T^	*Prunus armeniaca*	China	Endophyte	OQ615269	OQ592540	OQ613285	OQ613313
*Ap. armeniaca*	SAUCC DL1844	*Prunus armeniaca*	China	Endophyte	OQ615268	OQ592539	OQ613284	OQ613312
*Ap. arctoscopi*	KUC21331^T^	Egg of *Arctoscopus japonicus*	Republic of Korea	–	MH498449	MH498529	MH498487	MN868918
*Ap. arundinis*	GZCC 20-0116^T^	*Aspergillus flavus sclerotium*	USA	Saprobe/endophyte	MW478899	MW481720	MW522968	MW522952
*Ap. aseptata*	KUNCC 23-14169^T^	*Dicranopteris pedata*	China	Endophyte	OR590335	OR590341	OR634943	OR634949
*Ap. aurea*	CBS 244.83^T^	Air	Spain	–	KF144935	AB220251	KF144981	KF145023
*Ap. balearica*	CBS 145129^T^	Undetermined Poaceae	Spain	Saprobe	MK014836	MK014869	MK017975	NA
*Ap. babylonica*	SAUCC DL1841^T^	*Salix babylonica*	China	Endophyte	OQ615267	OQ592538	OQ613283	OQ613311
*Ap. babylonica*	SAUCC DL1864	*Salix babylonica*	China	Endophyte	OQ615266	OQ592537	OQ613282	OQ613310
*Ap. bambusicola*	MFLUCC 20-0144^T^	Culms of *Schizostachyum* *brachycladum*	Thailand	Saprobe	MW173087	MW173030	NA	MW183262
*Ap. bawanglingensis*	SAUCC BW0444^T^	*Indocalamus longiauritus*	China	–	OR739570	OR739429	OR757126	OR753446
*Ap. biserialis*	CGMCC 3.20135^T^	Bamboo	China	Saprobe	MW478885	MW481708	MW522955	MW522938
*Ap. camelliaesinensis*	LC 5007^T^	*Camellia sinensis*	China	Endophyte	KY494780	KY494704	KY705173	KY705103
*Ap. cannae*	ZHKUCC 22-0127	*Canna*	China	Saprobe	OR164948	NA	OR166321	OR166285
*Ap. chiangraiense*	MFLUCC 21-0053^T^	Dead culms of bamboo	Thailand	Saprobe	MZ542524	MZ542520	MZ546409	NA
*Ap. chromolaenae*	MFLUCC 17-1505^T^	*Chromolaena odorata*	Thailand	Saprobe	MT214436	MT214342	NA	NA
*Ap. cordylinae*	GUCC 10027^T^	*Cordyline fruticosa*	China	–	NA	MT040106	MT040148	MT040127
*Ap. coryli*	CFCC 58978^T^	*Corylus yunnanensis*	China	Saprobe	OR133586	OR125564	OR139978	OR139974
*Ap. cyclobalanopsidis*	CGMCC 3.20136^T^	*Cyclobalanopsidis glauca*	China	Saprobe	MW478892	MW481713	MW522962	MW522945
*Ap. dehongensis*	GMBCC1011^T^	Bamboo	China	Saprobe	PQ111483	PQ111494	PQ463974	PQ464025
*Ap. dehongensis*	GMBCC1012	Bamboo	China	Saprobe	PQ111484	PQ111495	PQ463975	PQ464026
*Ap. dematiacea*	KUNCC 23-14202^T^	*Dicranopteris ampla*	China	Endophyte	OR590339	OR590346	OR634948	OR634953
*Ap. descalsii*	CBS 145130^T^	*Ampelodesmos mauritanicus*	Spain	Saprobe	MK014837	MK014870	MK017976	NA
*Ap. dichotomanthi*	LC 4950^T^	*Dichotomanthes tristaniicarpa*	China	Saprobe/ endophyte	KY494773	KY494697	KY705167	KY705096
*Ap. dicranopteridis*	KUNCC 23-14171	*Dicranopteris pedata*	China	Endophyte	OR590336	OR590342	OR634944	OR634950
*Ap. dongyingensis*	SAUCC 0302^T^	On diseased leaves of bamboo	China	–	OP572424	OP563375	OP573270	OP573264
*Ap. elliptica*	ZHKUCC 22-0131	On dead stem of unidentified plant	China	Saprobe	OR164952	NA	OR166323	OR166284
*Ap. endophytica*	ZHKUCC 23-0006^T^	*Wurfbainia villosa*	China	Endophyte	OQ587984	OQ587996	OQ586075	OQ586062
*Ap. esporlensis*	CBS 145136^T^	*Phyllostachys aurea*	Spain	Saprobe	MK014845	MK014878	MK017983	NA
*Ap. euphorbiae*	IMI 285638b	*Bambusa* sp.	Bangladesh	Saprobe	AB220335	AB220241	AB220288	NA
*Ap. fermenti*	KUC 21289^T^	Seaweed	Republic of Korea	–	MF615213	MF615226	MF615231	MH544667
*Ap. fujianensis*	CGMCC 3.25647^T^	Diseased bamboo leaves	China	–	PP159034	PP159026	PP488470	PP488454
*Ap. fujianensis*	CGMCC 3.25648	Diseased bamboo leaves	China	–	PP159035	PP159027	PP488471	PP488455
*Ap. fuzhouensis*	CGMCC 3.25649^T^	Diseased bamboo leaves	China	–	PP159036	PP159028	PP488472	PP488456
*Ap. fuzhouensis*	CGMCC 3.25650	Diseased bamboo leaves	China	–	PP159037	PP159028	PP488473	PP488457
*Ap. gaoyouensis*	CFCC 52301^T^	Phragmites australis	China	Saprobe	NA	MH197124	MH236789	MH236793
*Ap. garethjonesii*	KUMCC 16-0202^T^	Dead culms of bamboo	China	Saprobe	KY356091	KY356086	NA	NA
*Ap. gelatinosa*	HKAS 11962^T^	Bamboo	China	Saprobe	MW478888	MW481706	MW522958	MW522941
*Ap. globosa*	KUNCC 23-14210^T^	*Dicranopteris linearis*	China	Endophyte	OR590340	OR590347	NA	OR634954
*Ap. globosa*	GMBCC1021	Bamboo	China	Saprobe	PQ111491	PQ111502	NA	PQ464027
*Ap. gongcheniae*	YNE00465^T^	Living stems of *Oryza meyeriana* subsp. *granulata*	China	Endophyte	PP033102	PP033259	PP034691	PP034683
*Ap. gongcheniae*	YNE00565	Living stems of *Oryza meyeriana* subsp. *granulata*	China	Endophyte	PP033103	PP0332560	PP034692	PP034684
*Ap.guangdongensis*	ZHKUCC 23-0004^T^	*Wurfbainia villosa*	China	Endophyte	OQ587982	OQ587994	OQ586073	OQ586060
*Ap. guangdongensis*	GMBCC1022	Bamboo	China	Saprobe	PQ111485	PQ111496	PQ463976	PQ464020
*Ap. guangdongensis*	GMBCC1023	Bamboo	China	Saprobe	PQ111486	PQ111497	PQ463977	PQ464021
*Ap. guiyangensis*	HKAS 102403^T^	Unidentified grass	China	Saprobe	MW240577	MW240647	MW775604	NA
*Ap. guizhouensis*	LC 5322^T^	Air in karst cave	China	Endophyte	KY494785	KY494709	KY705178	KY705108
*Ap. guizhouensis*	GZCC 20–0114	bamboo		–	MW478895	MW481716	MW522964	MW522948
*Ap. hainanensis*	SAUCC 1681^T^	On diseased leaves of bamboo	China	Pathogen	OP572422	OP563373	OP573268	OP573262
*Ap. hispanica*	IMI 326877^T^	Beach sand	Spain	Saprobe	AB220336	AB220242	AB220289	NA
*Ap. hydei*	CBS 114990^T^	Culms of *Bambusa* *tuldoides*	China	Saprobe/endophyte	KF144936	KF144890	KF144982	KF145024
*Ap. hyphopodii*	MFLUCC 15-0003^T^	*Bambusa tuldoides*	China	Saprobe	NA	KR069110	NA	NA
*Ap. hyphopodii*	KUMCC 16-0201	*Bambusa tuldoides*	China	Saprobe	KY356093	KY356088	NA	NA
*Ap. hysterina*	ICMP 6889^T^	Bamboo	New Zealand	Saprobe	MK014841	MK014874	MK017980	MK017951
*Ap. iberica*	CBS 145137^T^	*Arundo donax*	Portugal	Saprobe	MK014846	MK014879	MK017984	NA
*Ap. intestini*	CBS 135835^T^	Gut of a grasshopper	India	Saprobe	KR149063	KR011352	KR011350	KR011351
*Ap. italica*	CBS 145138^T^	*Arundo donax*	Italy	Saprobe	MK014847	MK014880	MK017985	MK017956
*Ap. jatrophae*	AMH 9557^T^	*Jatropha podagrica*	Italy	Saprobe	NA	JQ246355	NA	NA
*Ap. jiangxiensis*	LC 4577^T^	*Maesa* sp.	China	Endophyte	KY494769	KY494693	KY705163	KY705092
*Ap. jinanensis*	SAUCC DL1981^T^	On diseased leaves of *Bambusaceae* sp.	China	Endophyte	OQ615273	OQ592544	OQ613289	OQ613317
*Ap. jinanensis*	SAUCC DL2000	On diseased leaves of *Bambusaceae* sp.	China	Endophyte	OQ615272	OQ592543	OQ613288	OQ613316
*Ap. jinghongensis*	GMB-W1013^T^	Bamboo	China	Saprobe	PQ140163	PQ140160	PQ463971	PQ464022
*Ap. jinghongensis*	GMB-W1014	Bamboo	China	Saprobe	PQ140164	PQ140161	PQ463972	PQ464023
*Ap. kogelbergensis*	CBS 113333 K	Dead culms of Restionaceae	South Africa	Saprobe	KF144938	KF144892	KF144984	KF145026
*Ap. koreana*	KUC21332^T^	Egg of *Arctoscopus japonicus*	Republic of Korea	–	MH498444	MH498524	MH498482	MH544664
*Ap. koreana*	KUNCC23-15553	*Bamboo* sp.	China	Saprobe	PP584787	PP584690	PP982289	PP933195
*Ap. lageniformis*	KUC21686^T^	Branch of *Phyllostachys pubescens*	Republic of Korea	–	ON787761	ON764022	ON806636	ON806626
*Ap. locuta-pollinis*	LC 11683^T^	*Brassica campestris*	China	Saprobe	NA	MF939595	MF939622	MF939616
*Ap. longistroma*	MFLUCC 11-0481^T^	Dead culms of bamboo	Thailand	Saprobe	KU863129	KU940141	NA	NA
*Ap. lophatheri*	CFCC 58975^T^	On diseased leaves of Lophatherum gracile	China	–	OR133588	OR125566	OR139980	OR139970
*Ap. machili*	SAUCC 1175A–4^T^	*Machilus nanmu*	China	–	OR739574	OR739433	OR757130	OR753450
*Ap. magnispora*	ZHKUCC 22-0001^T^	Bamboo	China	Saprobe	OM486971	OM728647	OM543544	OM543543
*Ap. malaysiana*	CBS 102053^T^	*Macaranga hullettii*	Malaysia	Saprobe	KF144942	KF144896	KF144988	KF145030
*Ap. marianiae*	AP18219^T^	*Phleum pratense*	Spain	Saprobe	ON692422	ON692406	ON677186	NA
*Ap. marii*	CBS 497.90^T^	Beach sands	Spain	Saprobe	KF144947	AB220252	KF144993	KF145035
*Ap. marina*	KUC21328^T^	Seaweed	Republic of Korea	–	MH498458	MH498538	MH498496	MH544669
*Ap. mediterranea*	IMI 326875	Air	Spain	Saprobe	AB220337	AB220243	AB220290	NA
*Ap. menglaensis*	KUNCC 24-17546^T^	Dead culms of bamboo	China	Saprobe	PP584790	PP584693	PP982292	PP933198
*Ap. menglaensis*	KUNCC 24-17547	Dead culms of bamboo	China	Saprobe	PP584791	PP584694	PP982293	PP933199
*Ap. minutispora*	17E-042^T^	Mountain soil	Republic of Korea	–	NA	LC517882	LC518888	LC518889
*Ap. montagnei*	AP301120^T^	Balearic Islands	Spain	Sprobes	ON692424	ON692408	ON677188	ON677182
*Ap. mori*	MFLU 18-2514^T^	*Morus australis*	China (Taiwan)	Saprobe	MW114393	MW114313	NA	NA
*Ap. mukdahanensis*	MFLUCC 21-0026^T^	Dead bamboo	Thailand	Saprobe	OP377742	OP377735	NA	OP381089
*Ap. multiloculata*	MFLUCC 21-0023^T^	Dead bamboo	Thailand	Saprobe	OL873138	OL873137	OL874718	NA
*Ap. mytilomorpha*	DAOM 214595^T^	*Andropogon* sp.	India	Saprobe	NA	KY494685	NA	NA
*Ap. ananasi*	MFLUCC 23-0101^T^	*Ananas comosus*	Thailand	Saprobe	OR438877	OR438410	OR538085	OR500339
*Ap. neobambusae*	LC 7106^T^	Leaves of bamboo	China	Saprobe/endophyte	KY494794	KY494718	KY705186	KY806204
*Ap. neochinense*	CFCC 53036^T^	*Fargesia qinlingensis*	China	Saprobe	NA	MK819291	MK818547	MK818545
*Ap. neogarethjonesii*	HKAS 102408^T^	Bamboo	China	Saprobe	MK070898	MK070897	NA	NA
*Ap. neogongcheniae*	YNE01248^T^	Living stems of Poaceae plant	China	Endophyte	PP033106	PP033263	PP034695	PP034687
*Ap. neogongcheniae*	YNE01260	Living stems of Poaceae plant	China	Endophyte	PP033107	PP033264	PP034696	PP034688
*Ap. neosubglobosa*	JHB 006	Bamboo	China	Saprobe	KY356094	KY356089	NA	NA
*Ap. neosubglobosa*	KUMCC 16-0203^T^	Bamboo	China	Saprobe	KY356095	KY356090	NA	NA
*Ap. obovata*	LC 4940^T^	*Lithocarpus* sp.	China	Endophyte	KY494772	KY494696	KY705166	KY705095
*Ap. oenotherae*	CFCC 58972^T^	On diseased leaves of *Oenothera bienni*	China	–	OR133590	OR125568	OR139982	OR139972
*Ap. olivata*	ZY22.052^T^	Soil	China	–	OR680598	OR680531	OR843234	OR858925
*Ap. olivata*	ZY22.053	Soil	China	–	OR680599	OR680532	OR843235	OR858926
*Ap. ovata*	CBS 115042^T^	*Arundinaria hindsii*	China	Saprobe	KF144950	KF144903	KF144995	KF145037
*Ap. paragongcheniae*	YNE00992^T^	Living stems of Poaceae plant	China	Endophyte	PP033104	PP033261	PP034693	PP034685
*Ap. paragongcheniae*	YNE01259	Living stems of Poaceae plant	China	Endophyte	PP033105	PP033262	PP034694	PP034686
*Ap. paraphaeosperma*	MFLUCC 13-0644^T^	Dead culms of bamboo	Thailand	Saprobe	KX822124	KX822128	NA	NA
*Ap. phragmitis*	CPC 18900	*Phragmites australis*	Italy	Saprobe	KF144956	KF144909	KF145001	KF145043
*Ap. phyllostachydis*	MFLUCC 18-1101^T^	*Phyllostachys heteroclada*	China	Saprobe	MH368077	MK351842	MK291949	MK340918
*Ap. piptatheri*	CBS 145149^T^	*Piptatherum miliaceum*	Spain	Saprobe	MK014860	MK014893	NA	NA
*Ap. pseudohyphopodii*	KUC21680^T^	Culm of *Phyllostachys pubescens*	Republic of Korea	–	ON787765	ON764026	ON806640	ON806630
*Ap. pseudoparenchymatica*	LC7234^T^	Leaves of bamboo	China	Endophyte	KY494819	KY494743	KY705211	KY705139
*Ap. pseudorasikravindrae*	KUMCC 20-0208^T^	*Bambusa dolichoclada*	China	Saprobe	NA	MT946344	MT947367	MT947361
*Ap. pseudosinensis*	CPC 21546^T^	On diseased leaves of bamboo	Netherlands	Saprobe	KF144957	KF144910	NA	KF145044
*Ap. pseudospegazzinii*	CBS 102052^T^	*Macaranga hullettii*	Malaysia	Saprobe	KF144958	KF144911	KF145002	KF145045
*Ap. pterosperma*	CPC 20193^T^	*Lepidosperma gladiatum*	Australia	Saprobe	KF144960	KF144913	KF145004	KF145046
*Ap. pusillisperma*	KUC21321^T^	Seaweed	Republic of Korea	–	MH498453	MH498533	MH498491	MN868930
*Ap. qinlingensis*	CFCC 52303^T^	*Fargesia qinlingensis*	China	Saprobe	NA	MH197120	MH236791	MH236795
*Ap. rasikravindrae*	LC 8179	*Brassica rapa*	China	Saprobe	KY494835	KY494759	KY705227	KY705155
*Ap. rasikravindrae*	MFLUCC 21-0051	Dead culms of bamboo	Thailand	Saprobe	MZ542527	MZ542523	MZ546412	MZ546408
*Ap. rasikravindrae*	MFLUCC 21-0054	Dead culms of maize	Thailand	Saprobe	MZ542526	MZ542522	MZ546411	MZ546407
*Ap. sacchari*	CBS 372.67	Air		Endophyte	KF144964	KF144918	KF145007	KF145049
*Ap. sacchari*	CBS 664.74	Soil under *Calluna vulgaris*	Netherlands	Endophyte	KF144965	KF144919	KF145008	KF145050
*Ap. saccharicola*	CBS 831.71	Air	Netherlands	Endophyte	KF144969	KF144922	KF145012	KF145054
*Ap. sargassi*	KUC21228^T^	*Sargassum fulvellum*	Republic of Korea	–	KT207696	KT207746	KT207644	MH544677
*Ap. sasae*	CBS 146808^T^	Dead culms	China	Saprobe	MW883797	MW883402	MW890120	NA
*Ap. septata*	CGMCC 3.20134^T^	Bamboo	China	Saprobe	MW478890	MW481711	MW522960	MW522943
*Ap. senecionis*	KUNCC23-15556^T^	*Senecio scandens*	China	Saprobe	PP584794	PP584697	NA	PP993513
*Ap. senecionis*	KUNCC23-15557	*Senecio scandens*	China	Saprobe	PP584795	PP584698	NA	PP993514
*Ap. serenensis*	IMI 326869^T^	Excipients, atmosphere, and home dust	Spain	Saprobe	AB220344	AB220250	AB220297	NA
*Ap. setariae*	CFCC 54041	*Setaria viridis*	China	Saprobe	NA	MT492004	MT497466	MW118456
*Ap. setostroma*	KUMCC 19-0217	Dead branches of bamboo	China	Saprobe	MN528011	MN528012	NA	MN527357
*Ap. shangrilaensis*	GMBCC1019^T^	Bamboo	China	Saprobe	PQ111481	PQ111492	PQ164976	PQ164974
*Ap. shangrilaensis*	GMBCC1020	Bamboo	China	Saprobe	PQ111482	PQ111493	PQ164977	PQ164975
*Ap. sichuanensis*	HKAS 107008^T^	Dead culm of grass	China	Saprobe	MW240578	MW240648	MW775605	NA
*Ap. sorghi*	URM 93000^T^	*Sorghum bicolor*	Brazil	Endophyte	NA	MK371706	MK348526	NA
*Ap.* sp.	ZHKUCC 23-0010	*Wurfbainia villosa*	China	Endophyte	OQ587988	OQ588000	OQ586079	OQ586066
*Ap.* sp.	ZHKUCC 23-0011	*Wurfbainia villosa*	China	Endophyte	OQ587989	OQ588001	OQ586080	NA
*Ap. stipae*	CBS 146804^T^	Dead culm of *Stipa gigantea*	Spain	Saprobe	MW883798	MW883403	MW890121	MW890082
*Ap. subglobosa*	MFLUCC 11-0397^T^	Dead culms of bamboo	Thailand	Saprobe	KR069113	KR069112	NA	NA
*Ap. subglobosa*	GMB-W1024	Bamboo	China	Saprobe	PQ140165	PQ140162	PQ463973	PQ464024
*Ap. subrosea*	LC 7292^T^	Leaves of bamboo	China	Endophyte	KY494828	KY494752	KY705220	KY705148
*Ap. taeanensis*	KUC21322^T^	Seaweed	Republic of Korea	–	NA	MH498515	MH498473	MH544662
*Ap. thailandica*	MFLUCC 15-0202^T^	Dead culms of bamboo	Thailand	Saprobe	KU863133	KU940145	NA	NA
*Ap. trachycarpi*	KUNCC23-15558^T^	*Trachycarpus fortune*	China	Saprobe	PP584798	PP584701	PP982298	PP933204
*Ap. trachycarpi*	KUNCC23-15559	*Trachycarpus fortune*	China	Saprobe	PP584799	PP584702	PP982299	PP933205
*Ap. tropica*	MFLUCC 21-0056^T^	Dead culms of bamboo	Thailand	Saprobe	OK491653	OK491657	NA	NA
*Ap. vietnamensis*	IMI 99670^T^	*Citrus sinensis*	Vietnam	Saprobe	KX986111	KX986096	KY019466	
*Ap. wurfbainiae*	ZHKUCC 23-0008^T^	*Wurfbainia villosa*	China	Endophyte	OQ587986	OQ587998	OQ586077	OQ586064
*Ap. xenocordella*	CBS 478.86^T^	Soil from roadway	Zimbabwe	–	KF144970	KF144925	KF145013	KF145055
*Ap. xenocordella*	CBS 595.66	On dead branches	Misiones	Saprobe	KF144971	KF144926	NA	NA
*Ap. xishuangbannaensis*	KUMCC 21-0695^T^	The wing of *Rhinolophus pusillus*	China	–	OP363248	ON426832	OR025930	OR025969
*Ap. yunnana*	MFLUCC 15-1002^T^	*Phyllostachys nigra*	China	Saprobe	KU863135	KU940147	NA	NA
*Ap. yunnanensis*	ZHKUCC 23-0014^T^	Grass	China	Saprobe	OQ587992	OQ588004	OQ586083	OQ586070
*Ap. zhaotongensis*	GMBCC1015^T^	Bamboo	China	Saprobe	PQ111489	PQ111500	PQ463980	PQ464016
*Ap. zhaotongensis*	GMBCC1016	Bamboo	China	Saprobe	PQ111490	PQ111501	PQ463981	PQ464017
*Ap. zhenxiongensis*	GMBCC1017^T^	Bamboo	China	Saprobe	PQ111487	PQ111498	PQ463978	PQ464018
*Ap. zhenxiongensis*	GMBCC1018	Bamboo	China	Saprobe	PQ111488	PQ111499	PQ463979	PQ464019
*Neoarthrinium urticae*	IMI 326344	Leaf litter	India	–	AB220339	AB220245	AB220292	NA

Newly generated sequences in this study are in bold. “T” indicates type materials; “NA” indicates information not available.

AMH, Ajrekar Mycological Herbarium, Pune, Maharashtra, India; CBS, Culture collection of the Westerdijk Fungal Biodiversity Institute, Utrecht, Netherlands; CFCC, China Forestry Culture Collection Center, Beijing, China; CGMCC, China General Micro biological Culture Collection; CPC, Culture collection of Pedro Crous, housed at the Westerdijk Fungal Biodiversity Institute; DAOM, Canadian Collection of Fungal Cultures, Ottawa, Canada; GMBCC, Guizhou Medical University Culture Collection, Guiyang, China; GMB-W, Herbarium of Guizhou Medical University, Guiyang, China; GUCC, Guizhou University Culture Collection, Guizhou, China; GZCC, Guizhou Culture Collection, China; HKAS, Herbarium of Cryptogams, Kunming Institute of Botany, Chinese Academy of Sciences, Yunnan, China; ICMP, International Collection of Microorganisms from Plants, New Zealand; IMI, Culture collection of CABI Europe UK Centre, Egham, UK; JHB, H.B. Jiang; KUC, the Korea University Fungus Collection, Seoul, Korea; SFC the Seoul National University Fungus Collection; KUMCC, Culture collection of Kunming Institute of Botany, Yunnan, China; KUNCC; Kunming Institute of Botany Culture Collection Center, Kunming, China; LC, Personal culture collection of Lei Cai, housed in the Institute of Microbiology, Chinese Academy of Sciences, China; MFLUCC, Mae Fah Luang University Culture Collection, Chiang Rai, Thailand; SAUCC, Shandong Agricultural University Culture Collection; URM, ZHKUCC, Zhongkai University of Agriculture and Engineering, Guangdong, China.

### Phylogenetic analyses

All newly obtained forward and reverse sequences were assembled using Geneious 9.1.2. Those assembled sequences were searched using BLASTn (http://blast.ncbi.nlm.nih.gov/, accessed on 16 January 2024) to retrieve the sequences of closely related strains. The preliminary identification results showed that our new collections match closest with *Apiospora*, then all available sequences of *Apiospora* were downloaded from the GenBank based on previous literature (**Table 2**). The matrix of consensus sequences was aligned with MAFFT v. 7 (Katoh and Standley, 2013). The sequence alignments were trimmed by using trimAl.v1.2rev59 [parameters: -gt 0.7 (ITS, *tub*2), -gt 0.8 (LSU), -gt 0.9 (*tef*1-*α*); Capella-Gutiérrez et al., 2009] and BioEdit v. 7.0 (Hall, 2004) to remove unclear and uninformative regions. The alignments of four genes (LSU, ITS, *tef*1-*α*, and *tub*2) were concatenated by Matrix 1.9 (Vaidya et al., 2011). The AliView 1.26 (Larsson, 2014) was used to convert Fasta files to Phylip (for Maximum likelihood) and Nexus (for Bayesian inference) formats. Maximum likelihood analyses (ML) were performed at the CIPRES web portal using RAxML-HPC2 on XSEDE (8.2.12) (Stamatakis et al., 2008; Stamatakis, 2014) with GTRGAMMA model with 1,000 bootstrap pseudoreplicates. Bayesian inference posterior probabilities (BYPP) (Zhaxybayeva and Gogarten, 2002) were evaluated by Markov Chain Monte Carlo (MCMC) in MrBayes on XSEDE (3.2.7a) (Rannala and Yang, 1996) in the CIPRES Science Gateway web (Ronquist et al., 2012). The model of nucleotide evolution was determined by MrMTgui (Ma, 2016); GTR+I+G was the best-fit model for the ITS and *tub*2, SYM+I+G was the best-fit model for the LSU, and GTR+G was the best-fit model for *tef*1-*α*. Six simultaneous Markov chains were run for 1,000,000 generations and trees were sampled at every 100th generation. Phylogenetic trees were viewed in FigTree v. 1.4.2 (http://tree.bio.ed.ac.uk/software/figtree/) (Rambaut, 2012) and formatted by using Adobe Illustrator CS v. 5.

### Registration of novel taxa

Newly introduced taxa were registered at the Index Fungorum (2024) and the identifiers were obtained, fulfilling the requirements as mentioned in Art. F.5.1 (International Code of Nomenclature for Algae, Fungi and Plant).

## Results

### Phylogenetic results

**Figure 1:**
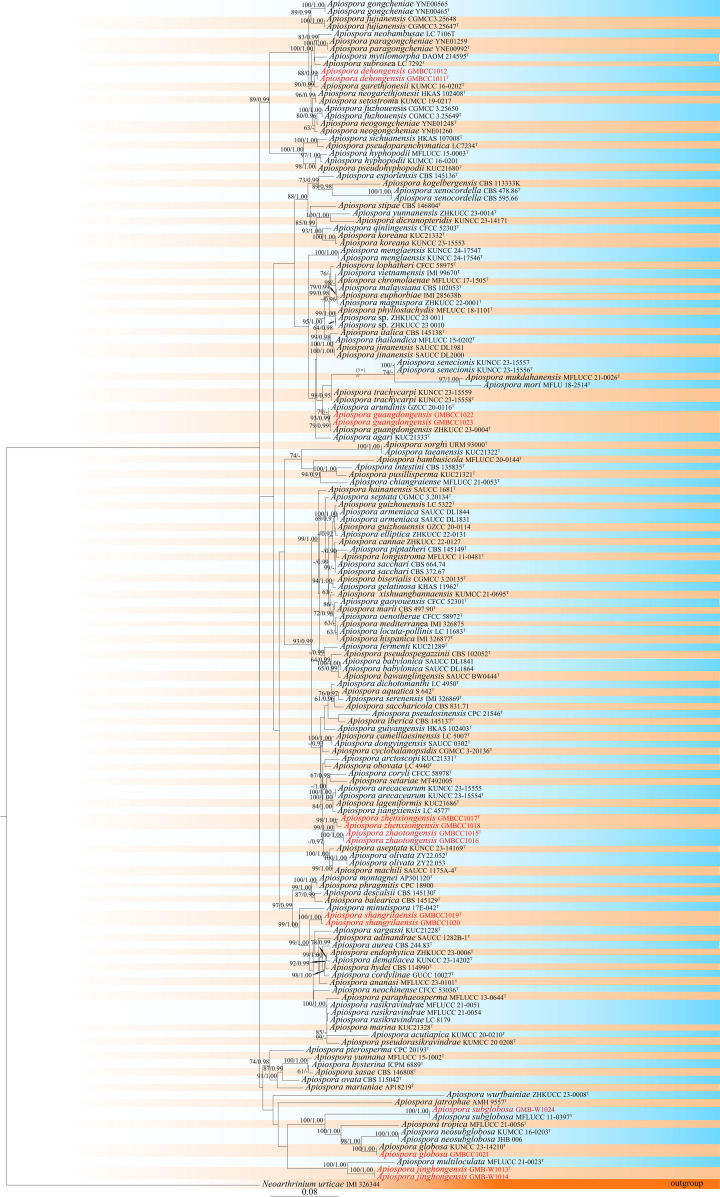
Phylogram retrieved from RAxML of *Apiospora* species using the combined dataset of LSU, ITS, *tef*1-*α*, and *tub*2 gene regions. The statistical values are provided at nodes as ML/PP (ML value equal to or above 60% and BI value equal to or above 0.90). The tree is rooted with *Neoarthrinium urticae* (IMI 326344). Ex-types and new strains are indicated by the superscript “T” and red respectively.

In this study, we selected 145 strains for the phylogenetic analysis and *Neoarthrinium urticae* (IMI 326344) as the outgroup taxon. In the phylogenetic analysis, the final alignment consisted of 2,099 characters in total, including gaps (ITS: 1–503 bp, LSU: 504–1,299 bp, *tef*1-*α*: 1,300–1,670 bp, *tub*2: 1,671–2,099 bp). The RAxML analysis of the combined dataset yielded the best scoring tree (**Figure 1**) with a final ML optimization likelihood value of −26,132.916506. The matrix had 1,073 distinct alignment patterns, with 14.23% undetermined characters or gaps. The estimated base frequencies were as follows: A = 0.231558, C = 0.249603, G = 0.260882, and T = 0.257956; substitution rates AC = 1.277568, AG = 3.415629, AT = 1.036259, CG = 0.978503, CT = 5.039154, and GT = 1.000000; and gamma distribution shape parameter α = 0.556683.

Furthermore, according to the phylogenetic results, two isolates (GMBCC1011 and GMBCC1012) have a close affinity to *Ap. garethjonesii* (KUMCC 16-0202, ex-type) with 90% ML and 0.99 BP bootstrap support. Two other isolates, GMBCC1022 and GMBCC1023, had a close affinity to *Ap. guangdongensis* (ZHKUCC 23-0004, ex-type). While GMBCC1019 and GMBCC1020 formed a distinct lineage with 100% ML bootstrap support and 1.00 posterior probability in BI analysis, GMBCC1017 and GMBCC1018 with GMBCC1015 and GMBCC1016 formed a sister branch with 99% ML and 1.00 BP statistical support. The isolate GMBCC1021 clustered with *Ap. globosa* (KUNCC 23-14210, ex-type) with 100% ML and 1.00 BP. The collection GMB-W1024 has a high similarity with *Ap. subglobosa* (MFLUCC 11-0397, ex-type) with 100% ML and 1.00 BP. Two isolates, GMB-W1013 and GMB-W1014, formed a distinct branch with *Ap. multiloculata* (MFLUCC 21-0023, ex-type) with 100% ML and 1.00 posterior probability in BI analysis (**Figure 1**).

### Taxonomic descriptions

*Apiospora* Sacc., Atti Soc. Veneto-Trent. Sci. Nat., Padova, Sér. 4 4: 85 (1875); Index Fungorum Registration Identifier: IF264; Classification: Apiosporaceae, Amphisphaeriales, Sordariomycetes.

**Figure 2:**
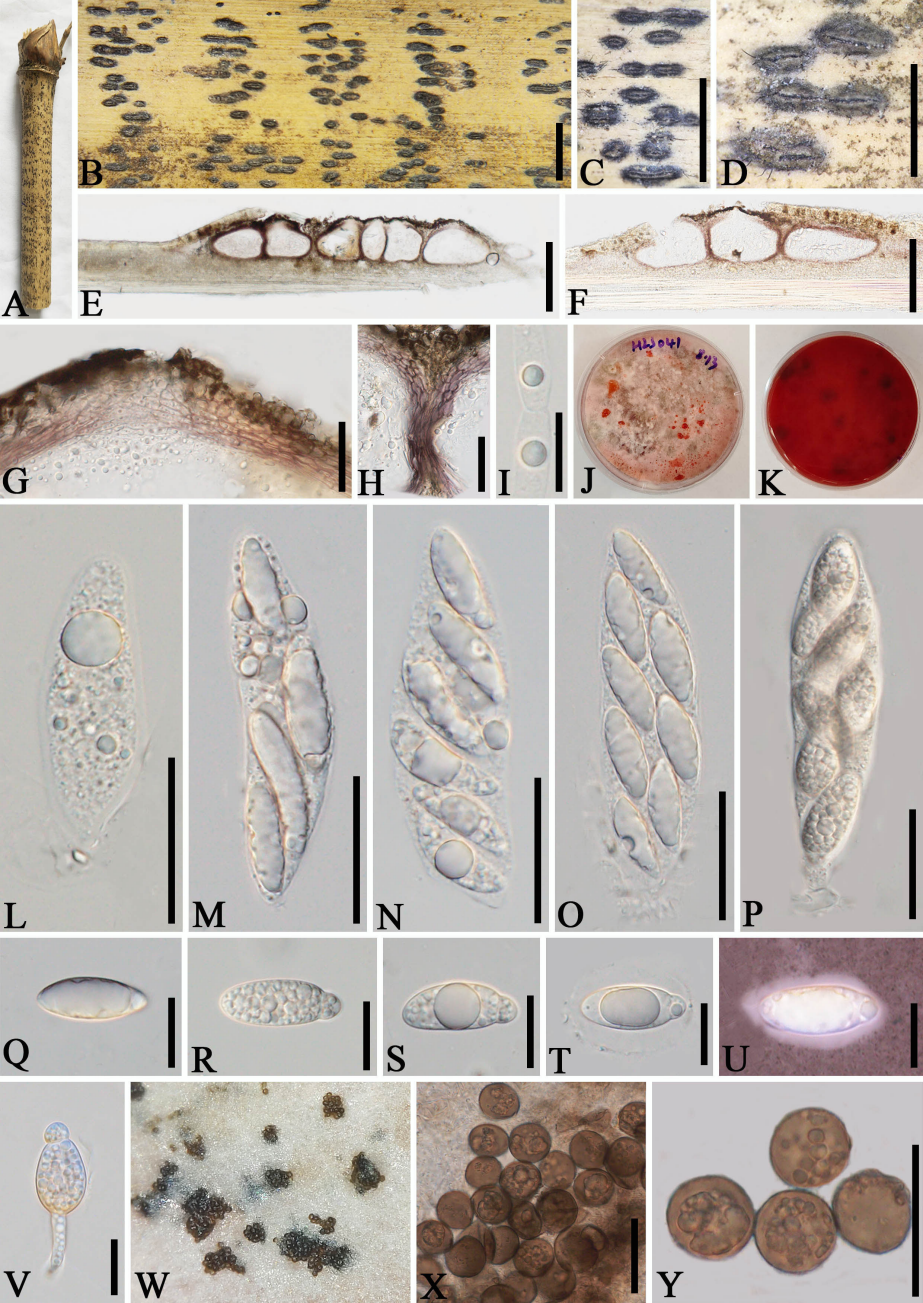
*Apiospora dehongensis* (GMB-W1011, holotype). **(A)** Bamboo specimen. **(B–D)** Stromata developing on bamboo branches. **(E, F)** Vertical sections of stromata. **(G, H)** Peridium. **(I)** Paraphyses. **(J, K)** Cultures on PDA with red pigmentation [upper **(J)**, reverse **(K)**]. **(L–P)** Asci. **(Q–T)** Ascospores. **(U)** Ascospore stained in Indian ink showing gelatinous sheath. **(V)** A germinating ascospore. **(W)** Conidia formed in culture. **(X, Y)** Conidia. Scale bars: **(B–D)** 2 mm, **(E)** 200 μm, **(F)** 150 μm, **(G, H, L–P, X, Y)** 30 μm, and **(I, Q–V)** 15 μm.

*Apiospora dehongensis* L.S. Han & D.Q. Dai, sp. nov. (**Figure 2**)

Index Fungorum Identifier: IF902463

Etymology: Named after the location “Dehong” where the new taxon was collected.

Description: *Saprobic* on dead branches of bamboo. Sexual morph: *Stromata* 0.4–2.5 mm long, 150–400 µm wide, 125–140 µm high, dark brown, fusiform or naviculate, raised on the host surface, with a slit-like opening at the top center, immersed, scattered to gregarious, or forming groups, uniloculate to multi-loculate. *Locules* 100–225 μm diameter × 95–130 μm high ( $\bar{\text{x}}$ = 145 × 105 µm, *n* = 20), gregarious, clustered, immersed in stromata, arranged in a row, obpyriform to subglobose, ostiolate at center with periphyses, membranous. *Peridium* 5–25 μm wide, composed of brown to purple to hyaline cells of *textura angularis*. *Hamathecium* 2.5–6.5 μm wide, filamentous, septate, unbranched, constricted at the septum. *Asci* 85–110 × 14–20 μm ( $\bar{\text{x}}$ = 97.5 × 17.5 μm, *n* = 20), 6-(8)-spored, unitunicate, clavate, apedicellate, apically rounded, straight to slight curved. *Ascospores* 25–30 × 10–12 μm ( $\bar{\text{x}}$ = 27 × 10.8 μm, *n* = 20), 1–3-seriate, ellipsoidal, 2-celled, with a large upper cell and a smaller lower cell, with guttules, hyaline, smooth-walled, with a gelatinous sheath. Asexual morph: Conidiophores and conidiogenous cells were not observed. *Conidia* forming on culture, 13–17.5 µm ( $\bar{\text{x}}$ = 16 µm, *n* = 20), globose to subglobose, dark brown, unicellular, smooth-walled, with guttules.

Culture characteristics: Ascospores germinate on PDA within 24 h. Colonies reached 60 mm after 20 days at 27 °C. The colonies are flat, white to reddish and produce red pigment on agar medium.

Material examined: CHINA, Yunnan Province, Dehong, Ruili, Jiexiang town (23°97′17″ N, 97°73′03″ E, 926 m), on dead branches of bamboo, 23 July 2023, L.S. Han & D.Q. Dai, HLS41 (GMB-W1011, holotype), ex-type GMBCC1011; *ibid*. (MHZU 24-0623, isotype), ex-isotype ZHKUCC 24-1160; *ibid.* HLS90 (GMB-W1012, isotype), ex-isotype GMBCC1012.

Notes: Phylogenetic analyses showed that newly generated strains GMBCC1011 and GMBCC1012 formed a sister branch to *Ap. garethjonesii* (D.Q. Dai & H.B. Jiang) Pintos & P. Alvarado (KUMCC 16-0202, ex-type) with 90% ML and 0.99 BI support (**Figure 1**). However, *tef*1-*α* and *tub*2 data of *Ap. garethjonesii* (KUMCC 16-0202, ex-type) are unavailable in GenBank. Morphologically, *Ap. dehongensis* differs from *Ap. garethjonesii* in having smaller asci (85–110 × 14–20 μm vs. 125–154 × 35–42 μm) and ascospores (25–30 × 10–12 μm vs. 30–42 × 11–16 μm) (Dai et al., 2016). Moreover, our cultures produced red pigment on PDA (**Figures 2J, K**), which was not observed in *Ap. garethjonesii* (Dai et al., 2016). Hence, based on morphological and culture characteristics and DNA sequence analyses, we introduce our new collection as a new species, *viz*., *Ap. dehongensis*.

**Figure 3:**
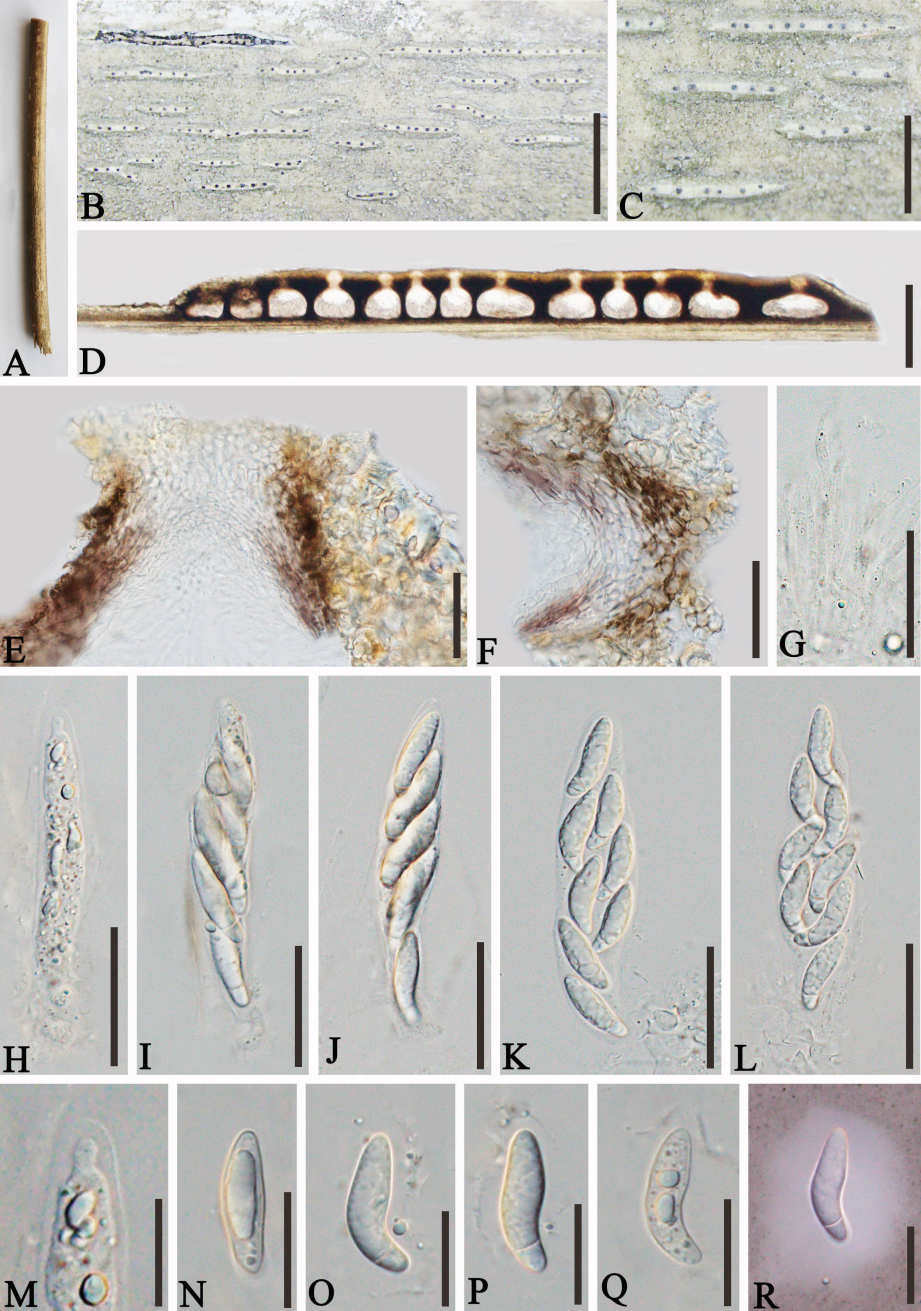
*Apiospora jinghongensis* (GMB-W1013, holotype). **(A)** Bamboo specimen. **(B, C)** Stromata developing on bamboo branches. **(D)** Vertical sections of stromata. **(E, F)** Peridium. **(G)** Paraphyses. **(H–L)** Asci. **(M)** Ascus with the rounded and smooth apex. **(N–R)** Ascospores [ascospore stained in Indian ink showing gelatinous sheath **(R)**]. Scale bars: **(B)** 2 mm, **(C)** 1 mm, **(D)** 300 μm, **(E–L)** 30 μm, and **(M–R)** 15 μm.

*Apiospora jinghongensis* L.S. Han & D.Q. Dai, sp. nov. (**Figure 3**)

Index Fungorum Identifier: IF902464

Etymology: Named after the location “Jinghong” where the new taxon was collected.

Description: *Saprobic* on dead branches of bamboo. Sexual morph: *Stromata* 0.6–5.5 mm long, 250–400 μm wide, 175–200 high, filiform, with parallel black spots, raised when mature, still under the host surface, scattered to gregarious, 2–20-loculate. *Locules* 110–240 μm diameter × 150–185 μm high ( $\bar{\text{x}}$ = 160 × 170 µm, *n* = 20), clustered, gregarious, immersed in stromata, forming groups, arranged in a row, ampulliform to obpyriform, usually with flattened base, brown to dark brown, membranous, with a periphysate ostiole in the center. *Peridium* 15–40 μm thick, composed of several layers, brown to hyaline cells of *textura angularis*. *Hamathecium* 1.5–4 μm wide, filamentous, hyaline, septate, unbranched paraphyses. *Asci* 85–105 × 13–20 μm ( $\bar{\text{x}}$ = 91.5 × 16 μm, *n* = 20), 8-spored, unitunicate, clavate to cylindrical, apically rounded, slightly curved, short pedicel. *Ascospores* 22–28 × 6.5–7.5 μm ( $\bar{\text{x}}$ = 23 × 6.7 μm, *n* = 20), biseriate, ellipsoidal, 1-septate, upper cell larger, and lower cell smaller, hyaline, smooth-walled, rounded at both ends, curved at the bottom, surrounded a gelatinous sheath. Asexual morph: Undetermined.

Material examined: CHINA, Yunnan Province, Xishuangbanna, Jinghong city, Manzhang, Mengla (21°91′97″ N, 101°20′42″ E, 617 m), on dead branches of bamboo, 16 August 2020, L.S. Han & D.Q. Dai, DDQ1033 (GMB-W1013, holotype); *ibid*. (MHZU 24-0624, isotype); *ibid.* DDQ1033-1 (GMB-W1014, isotype).

Notes: The phylogenetic tree shows that our new collections GMB-W1013 and GMB-W10134 formed a distinct sister branch to *Ap. multiloculata* Zhang et al. (MFLUCC 21-0023) with 100% ML and 1.00 BI support (**Figure 1**). Additionally, the nucleotide pairwise of new collections and *Ap. multiloculata* in ITS, LSU, and *tub*2 have 6.9% (37/535 bp), 0.8% (7/793 bp), and 9.7% (41/421 bp) differences, respectively. Morphologically, our new collection resembles *Ap. multiloculata* in having filiform stromata with central ostiole, 1-septate ascospores curved at the lower cell, but differs by having wider locules (110–240 μm vs. 88–160 μm) (Bhunjun et al., 2022). Although the new taxon morphologically resembles *Ap. multiloculata*, based on multigene phylogenetic analyses, we introduced *Ap. jinghongensis.*

**Figure 4:**
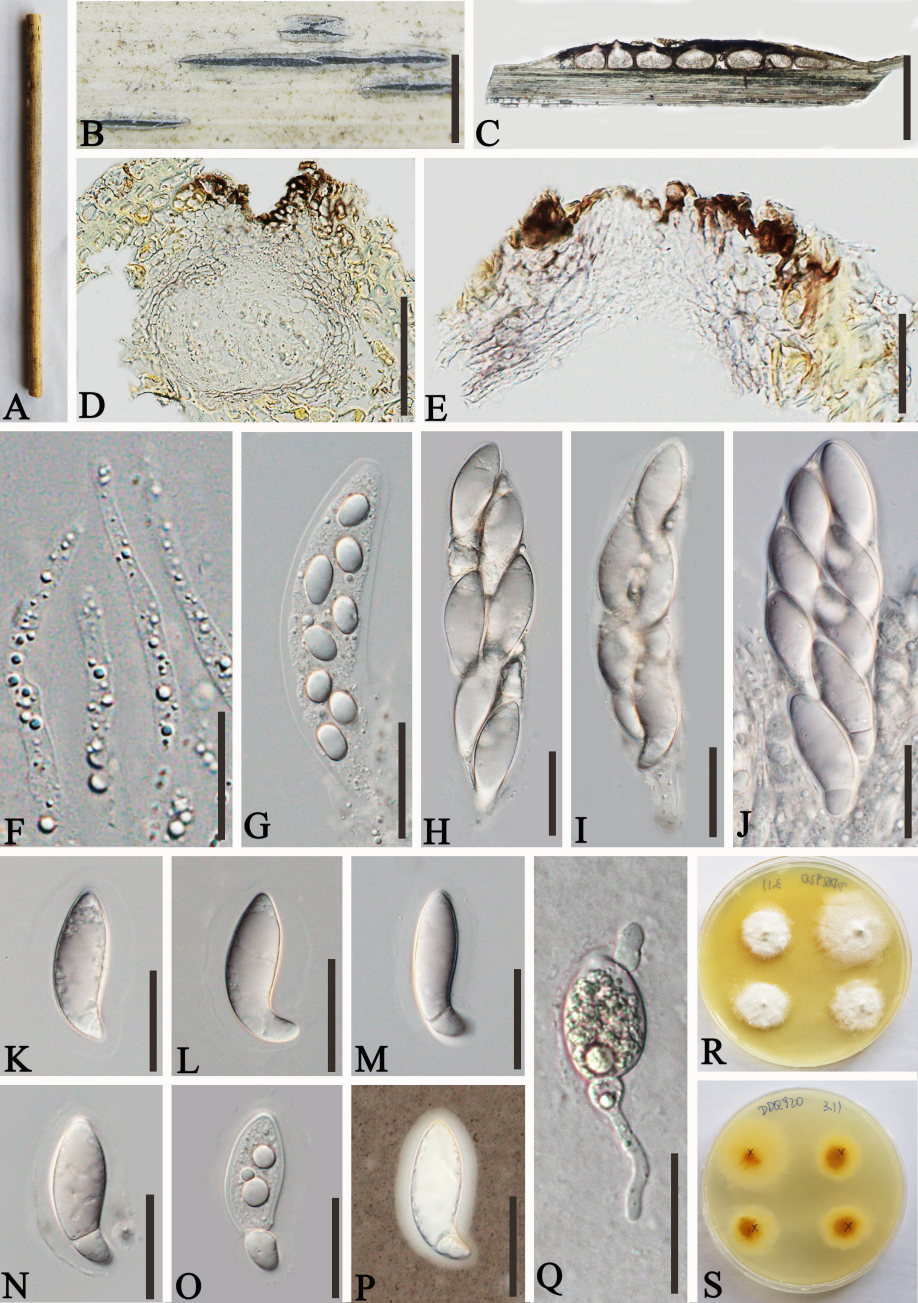
*Apiospora shangrilaensis* (GMB-W1019, holotype). **(A)** Bamboo specimen. **(B)** Stromata developing on bamboo branches. **(C, D)** Vertical sections of stromata. **(E)** Peridium. **(F)** Paraphyses. **(G–J)** Asci. **(K–P)** Ascospores [a ascospore stained in Indian ink showing gelatinous sheath **(P)**]. **(Q)** The germinating ascospore. **(R, S)** Cultures on PDA [upper **(R)**, reverse **(S)**]. Scale bars: **(C)** 500 μm, **(D, E, G–Q)** 50 μm, **(F)** 15 μm.

*Apiospora shangrilaensis* L.S. Han & D.Q. Dai, sp. nov. (**Figure 4**)

Index Fungorum Identifier: IF902465

Etymology: Named after the location “Shangri-La” where the new taxon was collected.

Description: *Saprobic* on dead culms of bamboo. Sexual morph: *Stromata* 1–3.2 mm long, 200–400 μm wide, 185–210 μm high, elongated fusiform, raised with long, black axis broken at the apex, immersed, multi-loculate. *Locules* 200–250 μm diameter × 120–190 μm high ( $\bar{\text{x}}$ = 228 × 157.5 µm, *n* = 20), gregarious, clustered, immersed in stromata, arranged in a row, ampulliform to subglobose with poor development base, pseudothecial, brown to dark brown. *Peridium* 10–25 µm wide, composed of three to five layers of hyaline to brown, cells of *textura angularis*. *Hamathecium* 2.5–4.5 µm wide, long, septate, slightly taping at the top, unbranched paraphyses. *Asci* 130–150 × 30–40 µm ( $\bar{\text{x}}$ = 140 × 36.6 µm, *n* = 20), 8-spored, unitunicate, clavata, apically rounded, straight to slightly curved, with short pedicel. *Ascospores* 40–55 × 14–17 µm ( $\bar{\text{x}}$ = 46.4 × 15.4 µm, *n* = 20), 1–2-seriate, ellipsoidal, aseptate when immature, 1-septate when mature, with a larger upper cell, and a smaller lower cell, occasionally with guttules, hyaline, smooth-walled, surrounded by a gelatinous sheath. Asexual morph: Undetermined.

Culture characteristics: Ascospores germinating on PDA within 24 h. Colonies reached 30 mm diameter in 20 days under dark and at 27°C conditions. Colonies flat, circular, cottony, irregular edge, white from above, yellow in the center and outward gradually becoming pale yellow to white from below.

Materials examined: CHINA, Yunnan Province, Shangri-La City, Bigu mountain (27°36′56.9″N, 99°42′6.4″E, 3,460 m), on dead culms of bamboo, 21 July 2020, L.S. Han & D.Q. Dai, DDQ00801 (GMB-W1019, holotype), ex-type, GMBCC1019, *ibid*. (MHZU 24-0625, isotype), ex-isotype ZHKUCC 24-1161, *ibid.* DDQ00920 (GMB-W1020), living culture GMBCC1020.

Notes: In the phylogenetic analyses, our new isolates, GMBCC1019 and GMBCC1020, formed a distinct branch (**Figure 1**). Morphologically, the new species is resembling *Ap. hydei* (Crous) Pintos & P. Alvarado (CBS 114990, ex-type) in having immersed, multi-loculate ascostromata, unitunicate asci, 1–septate, smooth-walled ascospores with a gelatinous sheath. However, our new collections can be distinguished from *Ap. hydei* (CBS 114990, ex-type) by having longer and wider asci (130–150 × 30–40 µm vs. 110–130 × 17–24 µm) and wider ascospores (40–55 × 14–17 µm vs. 35–45 × 8.5–11 µm) (Dai et al., 2016; Zeng et al., 2022). Hence, we introduced *Ap. shangrilaensis* as a new member of *Apiospora* based on morphological characteristics and phylogeny.

**Figure 5:**
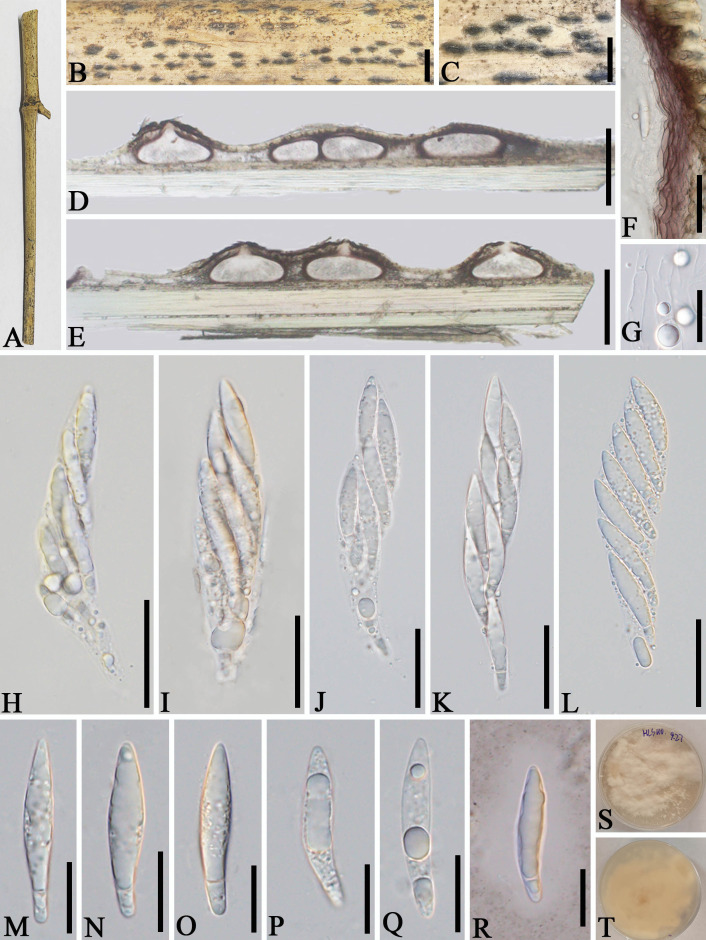
*Apiospora zhaotongensis* (GMB-W1015, holotype). **(A)** Bamboo specimen. **(B, C)** Stromata developing on bamboo branches. **(D, E)** Vertical sections of stromata. **(F)** Peridium. **(G)** Paraphyses. **(H–L)** Asci. **(M–R)** Ascospores. **(R)** The ascospore stained in Indian ink showing gelatinous sheath. **(S, T)** Cultures on PDA [upper **(S)**, reverse **(T)**]. Scale bars: **(B)** 1.5 mm, **(C)** 1 mm, **(D, E)** 300 μm, **(F, H–L)** 30 μm, and **(G, M–R)** 15 μm.

*Apiospora zhaotongensis* L.S. Han & D.Q. Dai, sp. nov. (**Figure 5**)

Index Fungorum Identifier: IF902466

Etymology: Named after the location “Zhaotong” where the new taxon was discovered.

Description: *Saprobic* on dead branches of bamboo. Sexual morph: *Stromata* 55–270 μm long, 250–450 μm wide, 140–180 μm high, naviculate or filiform, raised but still under on the host tissue with a slit-like opening at the top, scattered to gregarious, uniloculate to multi-loculate, black. *Locules* 150–290 diameter × 100–170 μm high ( $\bar{\text{x}}$ = 250 × 130 µm, *n* = 20), immersed in stromata, scattered or clustered, dark brown to black, obpyriform to subglobose, with a central ostiole, papillate. *Peridium* 25–45 μm thick, composed of several layers, dark brown to hyaline cells of *textura angularis*. *Hamathecium* 2.5–4.5 μm wide, filamentous distinctly septate, constricted at the septum, unbranched paraphyses, with guttules. *Asci* 85–110 × 14–20 μm ( $\bar{\text{x}}$ = 97.5 × 17.5 μm, *n* = 20), 8-spored, unitunicate, cylindrical, straight to slightly curved, apically rounded, apedicellate. *Ascospores* 30–40 × 6–7.5 μm ( $\bar{\text{x}}$ = 36 × 6.5 μm, *n* = 20), overlapping, 2-seriate, 1-sepetate, conical at both ends, with a larger upper cell and a smaller lower cell, some with guttules, hyaline, smooth-walled, mostly straight, sometimes slightly curved, surrounded by a gelatinous sheath. Asexual morph: Undetermined.

Culture characteristics: Ascospores germinate on PDA within 24 h. Colonies reached 60 mm after 20 days at 27°C. The colonies are white, fluffy, cottony, with irregular edge.

Material examined: CHINA, Yunnan Province, Zhaotong City, Zhenxiong Town (27°62′52″N, 104°81′98″E), on dead branches of bamboo, 4 August 2023, L.S. Han & D.Q. Dai, HLS110 (GMB-W1015, holotype), ex-type GMBCC1015; *ibid*. (MHZU 24-0626, isotype), ex-isotype, ZHKUCC 24-1162; *ibid.* HLS110-1 (GMB-W1016, isotype), GMBCC1016 (ex-isotype).

Notes: Two newly generated strains, GMBCC1015 and GMBCC1016, are phylogenetically close to *Ap. zhenxiongensis* (GMBCC1017 ex-type, GMBCC1018) (**Figure 1**). Morphologically, the new taxon can be distinguished from *Ap. zhenxiongensis* in having ascospores conical at both ends, whereas *Ap. zhenxiongensis* ascospores are rounded at both ends. Moreover, *Ap. zhaotongensis* has straighter ascospores at the bottom than *Ap. zhenxiongensis.* Therefore, we introduce a novel species, *Ap. zhaotongensis*, to accommodate our new collection based on morphology and phylogeny.

**Figure 6:**
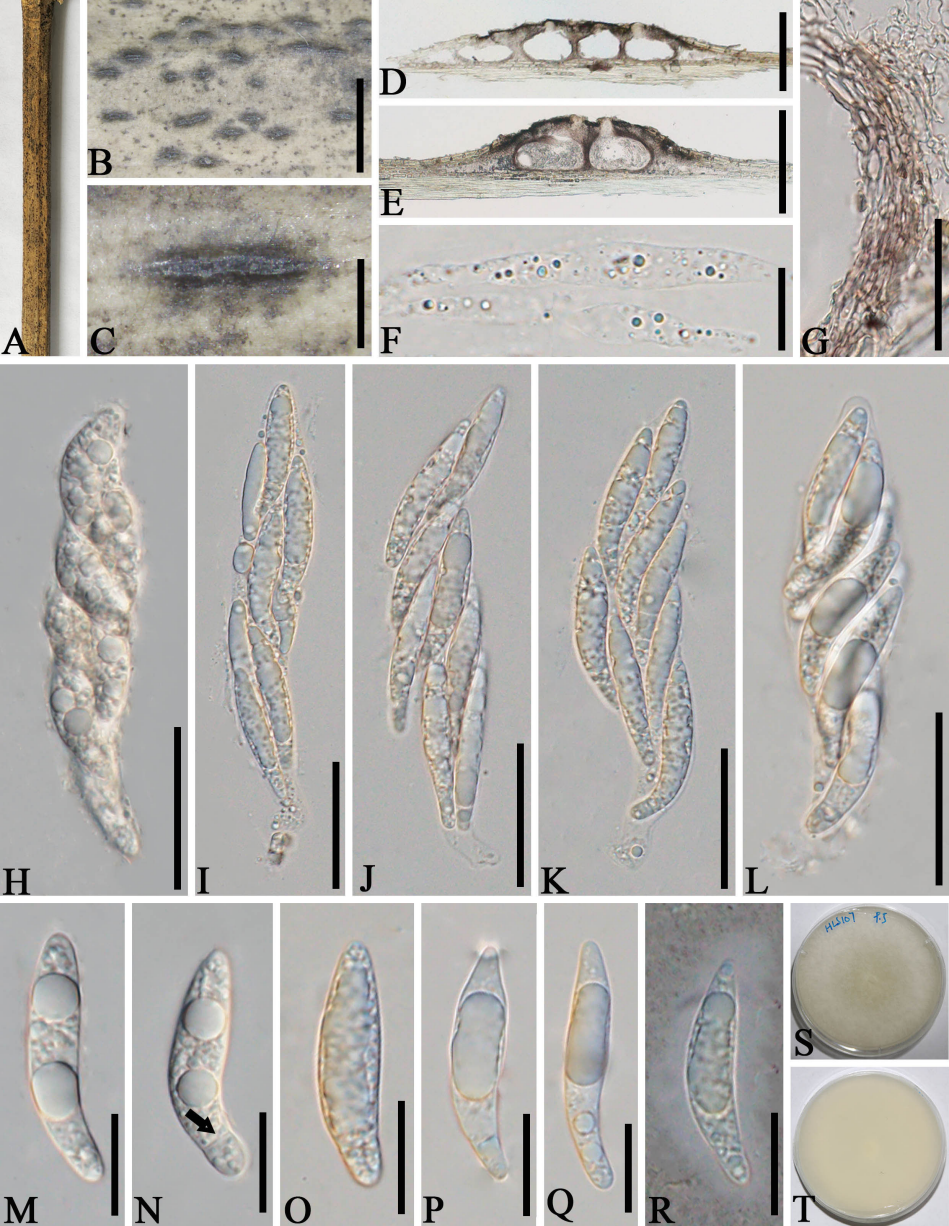
*Apiospora zhenxiongensis* (GMB-W1017, holotype). **(A)** Bamboo specimen. **(B, C)** Stromata developing on bamboo branches. **(D, E)** Vertical sections of stromata. **(F)** Paraphyses. **(G)** Peridium. **(H–L)** Asci. **(M–R)** Ascospores [a ascospore stained in Indian ink showing gelatinous sheath **(R)**]. **(S, T)** Cultures on PDA [upper **(S)**, reverse **(T)**]. Scale bars: **(B)** 1.5 mm, **(C)** 500 μm, **(D, E)** 300 μm, **(F, M–R)** 15 μm, and **(G, H–L)** 30 μm.

*Apiospora zhenxiongensis* L.S. Han & D.Q. Dai, sp. nov. (**Figure 6**)

Index Fungorum Identifier: IF902467

Etymology: Named after the location “Zhenxiong” where the new taxon was discovered.

Description: *Saprobic* on dead branches of bamboo. Sexual morph: *Stromata* 0.45–1.6 mm long, 200–450 μm wide, 140–160 μm high, raised, with a slit-like opening at the top, dark brown to black, scattered to gregarious, naviculate or filiform, multi-loculate. *Locules* 130–230 μm diameter × 90–150 μm high ( $\bar{\text{x}}$ = 183 × 128 µm, *n* = 20), immersed in stromata, arranged in a row, obpyriform to ampulliform, dark brown to black. *Ostiole* 30–60 µm wide, 35–65 µm high, with a black papillate. *Peridium* 5–25 μm thick, composed of several layers of brown cells of *textura angularis*. *Hamathecium* 3–8 μm wide, hyaline, septate, unbranched paraphyses. *Asci* 80–110 × 15–25 μm ( $\bar{\text{x}}$ = 95 × 19 μm, *n* = 20), 8-spored, unitunicate, cylindrical, apically rounded, with short pedicel. *Ascospores* 30–40 × 6–8.5 μm ( $\bar{\text{x}}$ = 34 × 7.5 μm, *n* = 20), overlapping, biseriate, ellipsoidal, rounded at both ends, 1-sepetate, cell above septa larger than those below, with guttules, hyaline, smooth-walled, distinctly curved at lower cell when mature, with a gelatinous sheath. Asexual morph: Undetermined.

Culture characteristics: Ascospores germinate on PDA within 24 h. Colonies reached 60 mm after 20 days at 27°C. The colonies are flat, white from above and below, dense, circular, cottony, with regular edge.

Material examined: CHINA, Yunnan Province, Zhaotong City, Zhenxiong (27°63′28″ N, 104°81′85″ E, 1,559 m), on dead branches of bamboo, 4 August 2023, L.S. Han & D.Q. Dai, HLS107 (GMB-W1017, holotype), ex-type GMBCC1017; *ibid*. (MHZU 24-0627, isotype), ex-isotype, ZHKUCC 24-1163; *ibid.* HLS136 (GMB-W1018), living culture GMBCC1018.

Notes: In our phylogenetic analyses, GMBCC1017 and GMBCC1018 formed a sister branch to *Ap. zhaotongensis* (GMBCC1015, ex-type and GMBCC1016) (99% ML, 1.00 BP, **Figure 1**). Morphologically, the new collections can be distinguished from *Ap. zhaotongensis* in having ascospores with rounded ends at both ends, while *Ap. zhaotongensis* ascospores have conical ends. Moreover, the ascospores of the new isolate are more curved at the lower cell than *Ap. zhaotongensis*. Hence, we introduced *Ap. zhenxiongensis* to accommodate our new collections based on morphological comparisons coupled with molecular data.

**Figure 7:**
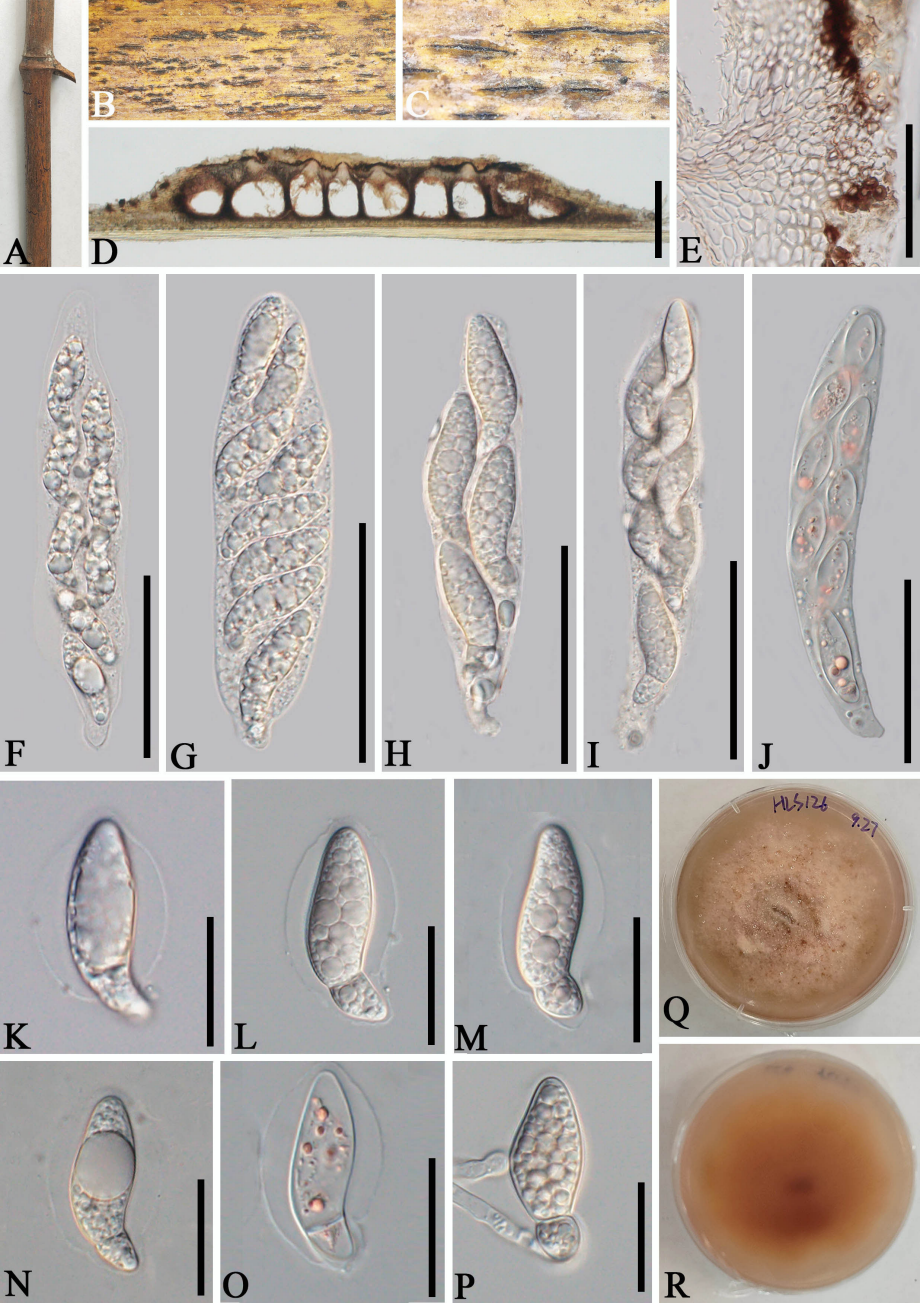
*Apiospora globosa* (GMB-W1021) **(A)** Bamboo specimen. **(B, C)** Stromata developing on bamboo branches. **(D)** Vertical sections of stromata. **(E)** Peridium. **(F–J)** Asci. **(K–O)** Ascospores surrounded by a gelatinous sheath. **(P)** A germinating ascospore. **(Q, R)** Cultures on PDA [upper **(Q)**, reverse **(R)**]. Scale bars: **(D)** 300 μm, **(E–J)** 50 μm, and **(K–P)** 20 μm.

*Apiospora globosa* J.Y. Zhang & Y.Z. Lu, *Journal of Fungi* 9 (no. 1,096) (2023) (**Figure 7**)

Index Fungorum Identifier: IF 901402

Description: *Saprobic* on dead culms of bamboo. Sexual morph: *Stromata* 0.45–3.3 mm long, 200–300 µm wide, 260–300 µm high, brown to black, fusiform, with stromata breaking through raised cracks at the black center, immersed, gregarious, multi-loculate. *Locules* 180–255 μm diameter × 100–240 μm high ( $\bar{\text{x}}$ = 222 × 164.5 µm, *n* = 20), gregarious, clustered, immersed in stromata, arranged in a row, obpyriform to ampulliform, ostiole with periphyses, membranous, brown to dark brown. *Peridium* 10–50 µm thick, composed of several layers of brown to hyaline, cells of *textura angularis*. *Asci* 100–135 × 21–25 µm ( $\bar{\text{x}}$ = 115.8 × 22.4 µm, *n* = 20), 4-(8)-spored, unitunicate, broadly cylindrical to clavate, with a short pedicel, straight to slightly curved, apically rounded. *Ascospores* 32–40 × 10–12.5 µm ( $\bar{\text{x}}$ = 34.6 × 11.3 µm, *n* = 20), 1–2-seriate, elliptical, 1–septate, with a larger upper cell, and a small lower cell, hyaline, with many guttules, smooth-walled, curved, constricted at the septum, surrounded by an entire gelatinous sheath. Asexual morph: *Endophytic* in the stems of *Dicranopteris linearis*, see Zhang et al. (2023a).

Culture characteristics: Ascospores germinate on PDA within 24 h. Colonies reached 55 mm after 20 days at 27°C. The colonies are white to pale reddish from above, pale reddish from below, circular, cottony, flat, spreading, with irregular edge.

Material examined: CHINA, Yunnan Province, Zhaotong City, Zhenxiong town (27°63′36″N, 104°81′84″E, 1,577 m), on dead culms of bamboo, 4 August 2023, L.S. Han & D.Q. Dai, HLS126 (GMB-W1021, first report of the sexual morph), living culture, GMBCC1021.

Notes: *Apiospora globos*a J.Y. Zhang & Y.Z. Lu was originally described by Zhang et al. (2023a) based on the asexual morph from a healthy stem of *Dicranopteris linearis* (KUNCC 23-14210, ex-type) collected from Guizhou Province, China. Our phylogenetic results (**Figure 1**) indicated that the strain GMBCC1021 is identical to the ex-type of *Ap. globosa* (KUNCC 23-14210, ex-type) with 100% MLBP and 1.00 BYPP statistic support. Moreover, the base pair arrangement of our collections with KUNCC 23-14210 is identical. Hence, in this study, we report the sexual morph of *Ap. globosa* for the first time.

**Figure 8:**
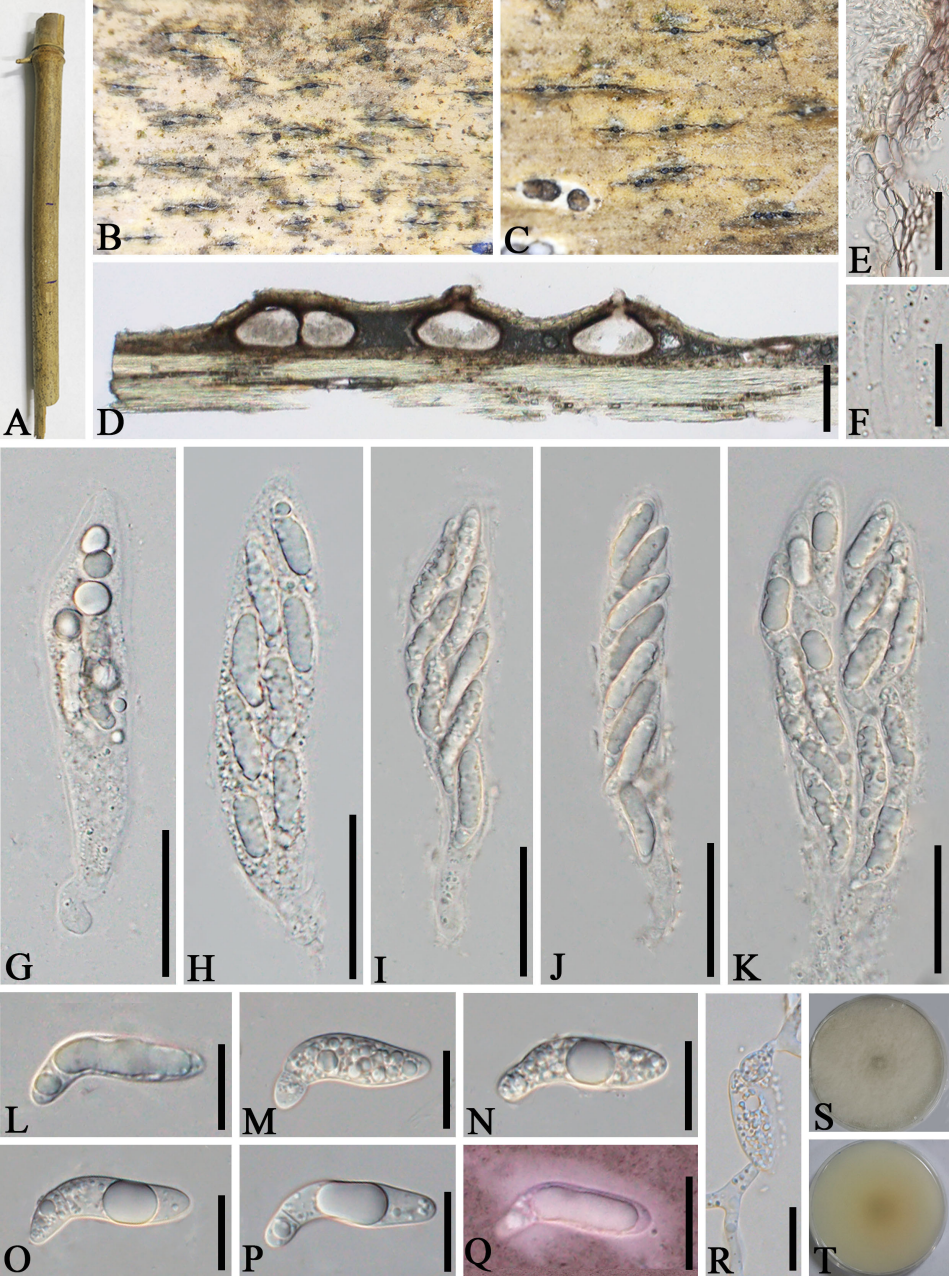
*Apiospora guangdongensis* (GMB-W1022) **(A)** Bamboo specimen. **(B, C)** Stromata developing on bamboo branches. **(D)** Vertical sections of stromata. **(E)** Peridium. **(F)** Paraphyses. **(G–K)** Asci. **(L–Q)** Ascospore [a ascospore stained in Indian ink showing gelatinous sheath **(Q)**]. **(R)** A germinating ascospore. **(S, T)** Cultures on PDA [upper **(S)**, reverse **(T)**]. Scale bars: **(D)** 150 μm, **(E, G–K)** 30 μm, **(F)** 10 μm, and (**L–R)** 15 μm.

*Apiospora guangdongensis* C.F. Liao & Doilom, *Journal of Fungi* 9 (no. 1,087): 12 (2023) (**Figure 8**)

Index Fungorum Identifier: IF 225951

Description: *Saprobic* on dead culms of bamboo. Sexual morph: *Stromata* 0.4–2.8 mm long, 250–350 mm wide, 130–190 μm high, raised on the host surface, with blackspots on slit-like opening, immersed, scattered to gregarious, 1–5-loculate, fusiform, brown. *Locules* perithecial, 210–380 μm diameter × 110–180 μm high ( $\bar{\text{x}}$ = 269 × 145 µm, *n* = 20), gregarious, clustered, immersed in stromata, arranged in a row, obpyriform to ampulliform to subglobose. *Ostiole* central, with periphyses. *Peridium* 5–25 μm wide, composed of dark brown to purple to hyaline cells of *textura angularis*. *Hamathecium* 2.5–4.5 μm wide, hyaline, septate, constricted at the septum, unbranched, not anastomosed paraphyses. *Asci* 90–120 × 16–21 μm ( $\bar{\text{x}}$ = 102 × 18 μm, *n* = 20), 8-spored, unitunicate, cylindrical, apically rounded, with a short pedicel, slightly curved. *Ascospores* 26–35 × 6.5–10 μm ( $\bar{\text{x}}$ = 31.5 × 8 μm, *n* = 20), biseriate, ellipsoidal, 2-celled, with a larger upper cell and a smaller lower cell, with guttules, hyaline, smooth-walled, rounded at both ends, with a gelatinous sheath.

Culture characteristics: Ascospores germinate on PDA within 24 h. Colonies reached 60 mm after 20 days at 27°C. The colonies are floccose, white, circular, cottony, with regular edge, no pigment.

Materials examined: CHINA, Yunnan Province, Zhaotong City, Zhenxiong town (27°63′28″ N, 104°81′88″ E, 1,557 m), on dead culms of bamboo, 4 August 2023, L.S. Han & D.Q. Dai, HLS51 (GMB-W1022, first report of the sexual morph), living culture GMBCC1022, GMB-W1023; *ibid.* HLS133 (GMB-W1023), living culture GMBCC1023.

Notes: Asexually typified, endophytic species, *Apiospora guangdongensis* C.F. Liao & Doilom (ZHKUCC 23-0004, ex-type) (from the leaves of *Wurfbainia villosa*) was originally described by Liao et al. (2023) from Guangdong Province, China. Our multi-gene phylogenetic tree (**Figure 1**) showed that our new isolates GMBCC1022 and GMBCC1023 grouped with *Ap. guangdongensis* (ZHKUCC 23-0004, ex-type). Moreover, the base pair arrangement of our collections with ZHKUCC 23-0004 was identical. Therefore, we reported the sexual morph of *Ap. guangdongensis* for the first time in this study.

**Figure 9:**
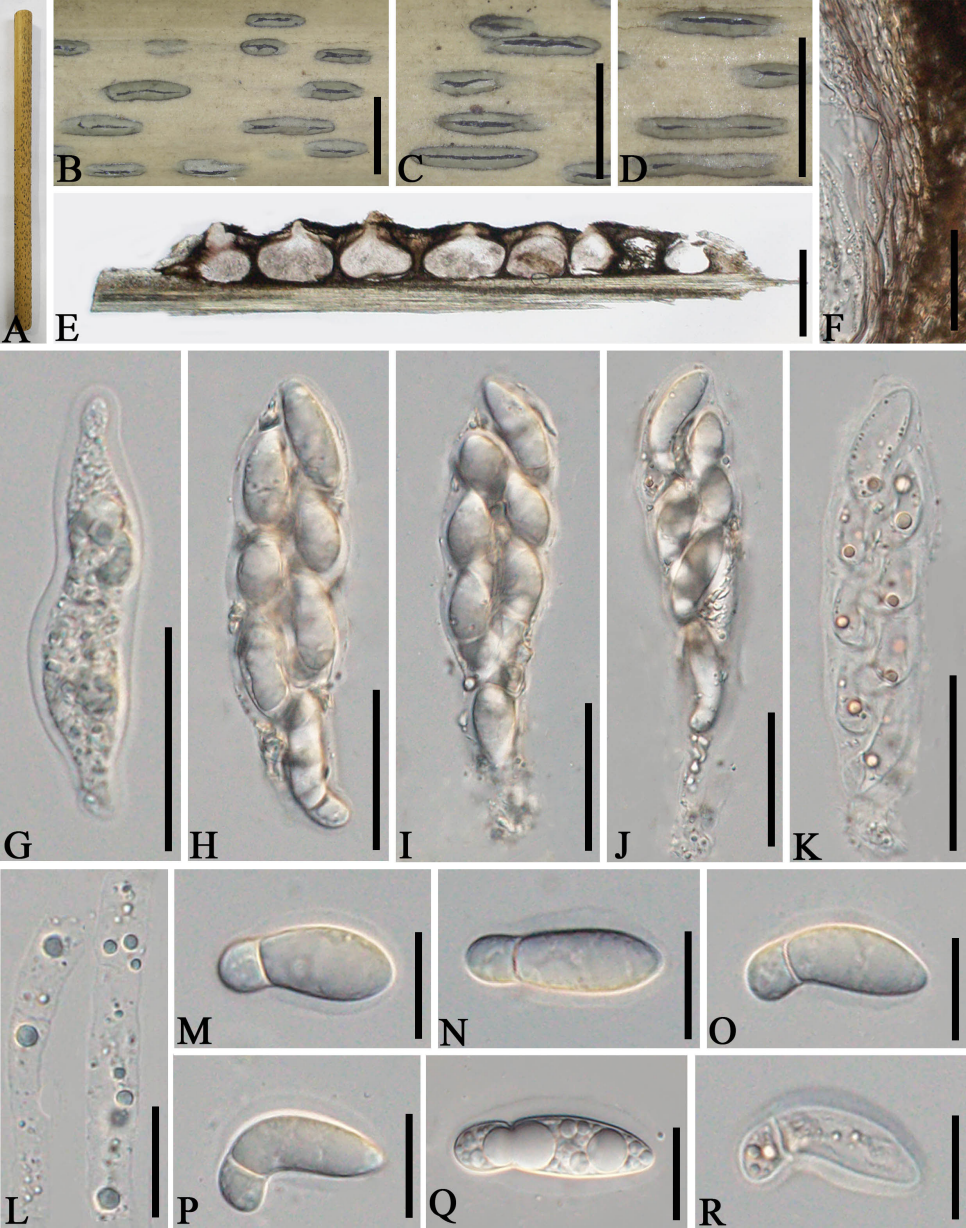
*Apiospora subglobosa* (GMB-W1024) **(A)** Bamboo specimen. **(B–D)** Stromata developing on bamboo branches. **(E)** Vertical sections of stromata. **(F)** Peridium. **(G–K)** Asci. **(L)** Paraphyses. **(M–R)** Ascospores surrounded by a gelatinous sheath. Scale bars: **(B–D)** 2 mm, **(E)** 300 μm, **(F–I, K)** 30 μm, and **(L–R)** 15 μm.

*Apiospora subglobosa* (D.Q. Dai & K.D. Hyde) Pintos & P. Alvarado, Fungal Systematics and Evolution 7: 207 (2021) (**Figure 9**)

Index Fungorum Identifier: IF 837715

See Senanayake et al. (2015) for the description.

Material examined: CHINA, Yunnan Province, Dehong, Mang City, Xuangang town (24°45′41″N, 98°43′83″E, 919 m), on dead culms of bamboo, 22 July 2023, L.S. Han & D.Q. Dai, HLS84 (GMB-W1024, new geographical record in China).

Known distributions: Thailand (Senanayake et al., 2015) and China (this study).

Known hosts: Bamboo (Senanayake et al., 2015, this study).

Notes: Senanayake et al. (2015) introduced *Arthrinium subglobosum* D.Q. Dai & K.D. Hyde, but later, Pintos and Alvarado (2021) transferred it to *Apiospora s. str.* as *Apiospora subglobosa* (D.Q. Dai & K.D. Hyde) Pintos & P. Alvarado. In our phylogenetic tree, our new collection GMB-W1024 clustered with *Ap. subglobosa* (MFLUCC 11-0397, ex-type) with 100% ML and 1.00 BI support (**Figure 1**). Morphologically, our new collection is similar to *Ap. subglobosa* in muti-loculate stromata with black slit-like opening, straight or curved, apical cell large, with smaller basal cell ascospores. However, the asci of GMB-W1024 are narrower than in MFLU 15-0384 (20–25 µm vs. 27–36 µm) (holotype). The ascospores of GMB-W1024 are longer than those of MFLU 15-0384 (25–33 µm vs. 24–28 µm) but narrower (7–10 μm vs. 8.5–12.5 μm), possibly due to the environmental change leading to slight differences in size. Nevertheless, we confirmed that our new collection (GMB-W1024) is *Ap. subglobosa* based on phylogenetic analyses (**Figure 1**).

## 4 Discussion

Fungal diversity in southwestern China is very high and a large number of species are introduced annually (Wijayawardene et al., 2021a; Lu et al., 2024; Du et al., 2023; Zhang et al., 2023b, 2024; Chen et al., 2024; Liu et al., 2023a; Tian et al., 2024; Xu et al., 2024; Zhang et al., 2024). Fungi associated with bamboo is one of the popular research topics among the mycologists in this region and several new species have been published in recent studies (e.g., *Bambusicola hongheensis fide* Phookamsak et al., 2024, *Parabambusicola yunnanensis fide* Han et al., 2023, *Paramphibambusa bambusicola fide* Han et al., 2024). However, a large number of taxa are yet to be discovered in this region and from bamboo plants, although it has been a well-studied host (Wijayawardene et al., 2021b, 2022).

In this study, we introduced five new species, *viz*., *Apiospora dehongensis*, *Ap. jinghongensis*, *Ap. shangrilaensis*, *Ap. zhaotongensis*, and *Ap. zhenxiongensis*, and two new sexual morph reports, *viz*., *Ap. globosa* and *Ap. guangdongensis*. Furthermore, one new geographical record of *Ap. subglobosa* was also reported based on morphological and multi-locus phylogenetic analyses. All the species were found as saprobic taxa on decaying bamboo branches and culms. Three specimens were collected from western Yunnan Province, China (Dehong), two specimens were obtained from the southwestern part (Xishuangbanna), seven specimens were collected from the northeastern section (Zhaotong), and two specimens were gathered from the northwestern part (Shangri-La), which displayed the highly hidden species richness of *Apiospora* in the different regions of Yunnan. Jiang et al. (2022b) and Wang et al. (2018) also emphasized the high species richness of bambusicolous ascomycetes in southwest China, with *Apiospora* as one of the genera with high species diversity. Thus, ongoing research on the genus *Apiospora* is essential.

According to the recently published studies, 90 species of *Apiospora* have been reported to have only an asexual morph, 19 species have been reported to have only a sexual morph, and 22 species have both morphs based on molecular data, including this study (Pintos and Alvarado, 2021; Li et al., 2023; Zhang et al., 2023a; Zhao et al., 2023; Ai et al., 2024; Dissanayake et al., 2024; Liu et al., 2024; Tian et al., 2024; Yan and Zhang, 2024; Zhao et al., 2024). Moreover, regarding the reports of *Apiospora* discovered on bamboo (based on molecular data), 17 species have been identified solely by their asexual morph, while 15 species have sexual morph only. However, 12 species have been reported with both morphs (Pintos and Alvarado, 2021; Liao et al., 2023; Zhao et al., 2023, 2024; Zhang et al., 2023a; Ai et al., 2024; Dissanayake et al., 2024). Therefore, it is necessary to continue studying bambusicolous *Apiospora* to explore more asexual or sexual morphs of known or unknown species. Furthermore, because some *Apiospora* species were reported to have only asexual or sexual forms, it is crucial to collect more specimens in nature to clarify the status of these species in the genus *Apiospora*.

Note that most of the *Apiospora* species reported from bamboo are saprobes, while only *Ap. dongyingensis* Liu et al. and *Ap. hainanensis* Liu et al. have been reported as pathogens (Liao et al., 2023; Liu et al., 2023b). Jiang et al. (2020) emphasized the importance of researching on bambusicolous pathogenic fungi, because pathogenic fungi have the potential to hinder the advancement of the bamboo industry and even lead to ecological problems. So far, more than 190 bambusicolous pathogenic fungi have been discovered (Kuai, 1996). The continuous study of bamboo pathogenic fungi is related to the conservation and utilization of bamboo resources, which is of great significance to the promotion of sustainable development. Thus, more search works on bambusicolous pathogenic fungi are needed.

## Data Availability

The datasets presented in this study can be found in online repositories. The names of the repository/repositories and accession number(s) can be found below: https://www.ncbi.nlm.nih.gov/genbank/, ITS: PQ140160, PQ140161, PQ140162, PQ111492, PQ111493, PQ111494, PQ111495, PQ111496, PQ111497, PQ111498, PQ111499, PQ111500, PQ111501, PQ111502; LSU: PQ140163, PQ140164, PQ140165, PQ111481,PQ111482, PQ111483, PQ111484, PQ111485, PQ111486PQ111487 PQ111488, PQ111489, PQ111490, PQ111491; *tub*2: PQ463971, PQ463972, PQ463973, PQ463974, PQ463975, PQ463976, PQ463977, PQ463978 PQ463979, PQ463980, PQ463981, PQ164976, PQ164977; *tef*1-*α*: PQ464016, PQ464017, PQ464018, PQ464019, PQ464020, PQ464021, PQ464022, PQ464023, PQ464024, PQ464025, PQ464026, PQ464027, PQ164974, PQ164975.
